# Validation of a cross-cultural instrument for child behavior problems: the Disruptive Behavior International Scale – Nepal version

**DOI:** 10.1186/s40359-018-0262-z

**Published:** 2018-11-03

**Authors:** Matthew D. Burkey, Ramesh P. Adhikari, Lajina Ghimire, Brandon A. Kohrt, Lawrence S. Wissow, Nagendra P. Luitel, Emily E. Haroz, Mark J. D. Jordans

**Affiliations:** 1 Department of Psychiatry, University of British Columbia, Vancouver, Canada; 2 Research Department, Helen Keller International Nepal, Lalitpur, Nepal; 3 Research Department, Transcultural Psychosocial Organization—Nepal, Kathmandu, Nepal; 4 Department of Psychiatry, George Washington University, Washington, DC USA; 5 Division of Child and Adolescent Psychiatry, Johns Hopkins School of Medicine, Baltimore, USA; 6 Research Department, Transcultural Psychosocial Organization—Nepal, Kathmandu, Nepal; 7 Research Department, War Child, and Amsterdam Institute for Social Science Research, University of Amsterdam, Amsterdam, Netherlands; 8 Department of Mental Health, Johns Hopkins Bloomberg School of Public Health, Baltimore, Maryland USA

**Keywords:** Disruptive behavior disorders, Oppositional defiant disorder, Conduct disorder, Child behavior problems, Externalizing disorders, Scale, Validation, Low-income countries, Nepal

## Abstract

**Background:**

Obtaining accurate and valid measurements of disruptive behavior disorders remains a challenge in non-Western settings due to variability in societal norms for child behavior and a lack of tools developed outside of Western contexts. This paper assesses the reliability and construct validity of the Disruptive Behavior International Scale – Nepal version (DBIS-N)—a scale developed using ethnographic research in Nepal—and compares it with a widely used Western-derived scale in assessing locally defined child behavior problems.

**Methods:**

We assessed a population-based sample of 268 children ages 5–15 years old in Nepal for behavior problems with a pool of candidate items developed from ethnographic research. We selected final items for the DBIS-N using exploratory factor analysis in a randomly selected half of the sample and then evaluated the model fit using confirmatory factor analysis in the remaining half. We compared the classification accuracy and incremental validity of the DBIS-N and Eyberg Child Behavior Inventory (ECBI) using local defined behavior problems as criteria. Local criteria were assessed via parent report using: 1) local behavior problem terms, and 2) a locally developed vignette-based assessment.

**Results:**

Ten items were selected for the final scale. The DBIS-N had good internal consistency (Cronbach’s α: 0.84) and excellent test-retest reliability (intraclass correlation 0.93, *r* = .93). Classification accuracy and area under the curve (AUC) were similar and high for both the ECBI (AUC: 0.83 and 0.85) and DBIS-N (AUC: 0.83 and 0.85) on both local criteria. The DBIS-N added predictive value above the ECBI in logistic regression models, supporting its incremental validity.

**Conclusions:**

While both the DBIS-N and the ECBI had high classification accuracy for local idioms for behavior problems, the DBIS-N had a more coherent factor structure and added predictive value above the ECBI. Items from the DBIS-N were more consistent with cultural themes identified in qualitative research, whereas multiple items in the ECBI that did not fit with these themes performed poorly in factor analysis. In conjunction with practical considerations such as price and scale length, our results lend support for the utility of the DBIS-N for the assessment of locally prioritized behavior problems in Nepal.

**Electronic supplementary material:**

The online version of this article (10.1186/s40359-018-0262-z) contains supplementary material, which is available to authorized users.

## Background

Behavior problems are among the most common childhood mental disorders worldwide [1, 2], and have substantial impacts on social, educational and psychological outcomes into adulthood [3]. With increasing efforts to measure and intervene upon mental disorders in low-income and non-Western settings, there is a need to evaluate the validity of disorder definitions and measurement tools that have primarily been developed in high-income, Western country settings [4, 5]. Scrutiny is particularly important in the case of child behavior problems, which are defined as patterns of violating *society-specific* norms for behavior [6]. Without careful contextual evaluation, there is a risk of pathologizing symptoms without perceived relevance or coherence in local settings, and of failing to identify children who may benefit from interventions [7]. This paper assesses the reliability and construct validity of a scale developed using ethnographic research in Nepal with a widely used Western-derived scale in assessing locally defined child behavior problems.

Valid assessment tools are needed in order to determine disorder prevalence, allocate limited resources, and appropriately target evidence-based treatment interventions [5]. Careful contextual adaptation is essential for mental health assessment tools given the variety of local behavioral concerns and the between-culture variability in normative affective and behavioral expectations [5]. An additional concern in using disorder definitions and tools developed in other cultural contexts is that of a “category fallacy”—that is, the risk of identifying clusters of symptoms that may have a substantially different meaning and/or association with impairment in the target context [7]. Cultural considerations may be especially important in the case of disruptive behavior disorders (DBDs), the definition of which (according to the Diagnostic and Statistical Manual of Mental Disorders, Fifth Edition (DSM-5)) depends on violation of *society-specific* norms for child behavior [6]. In addition to cross-cultural validity, there are important pragmatic limitations to using existing assessment tools in low-resource settings, including the cost of proprietary scales and the time required to complete lengthy assessments.

### Epidemiology and measurement issues for disruptive behavior problems

As one of the most common child mental disorders and important risk factors for academic failure, delinquency, and affective disorders [3], DBDs represent an important, but neglected, public health problem in low- and middle-income countries (LMIC). A large meta-analysis demonstrated consistent rates of Oppositional Defiant Disorder (ODD) and Conduct Disorder (CD) across geographic regions globally [1], though only two studies were included from LMIC [8, 9]. However, a more recent large-scale meta-analysis of child mental disorders [2] showed very high variability (*I*^*2*^ > 99%) in prevalence estimates of disruptive behavior disorders, suggesting possible measurement error across populations. Existing epidemiologic and treatment studies of DBDs have predominantly relied on diagnostic tools developed in the United States or Western Europe with minimal adaptation (usually limited to translation and back-translation) to the local context [10]. Consequently, the paucity of studies of DBDs in LMICs is compounded by uncertainty about the validity of their findings, and there is a shortage of useful clinical tools for identifying children in need of treatment for behavior problems.

Validation and cultural adaptation of assessment tools is important for child behavior problems given the wide variability in role and behavioral expectations for children between settings. DBDs are some of the few disorders for which DSM-5 makes special note of the importance of culture and context in determining variance in normative levels of symptoms [6]. In addition to varying normative levels of symptoms, the specific behaviors of concern (i.e. those that “bring the individual in conflict with societal norms or authority figures” [6]) vary widely between societies, by definition. For example, a qualitative study in Rwanda identified local conduct problems that were not easily categorized under DSM-5 symptoms. Key indicators of a local conduct problem (*ubarara*) in Rwanda included: “roaming around/moving without purpose”, “being independent/unruled”, “speaking rudely”, and “not being grateful for what is given to him/her” [11]. There are few other examples of cultural studies of child behavior problems in non-Western or LMIC settings.

Another key aspect of cross-cultural validity highlighted in the concept of category fallacy is the association of symptoms with impairment or distress. That is, symptoms (i.e. specific behaviors) may be manifested in different settings, but may not be seen as problematic to the same extent. For example, in a study employing case vignettes, Weisz et al. [12] demonstrated that Thai parents compared with U.S. parents rated behavior problems as less serious, less worrisome, and more likely to improve with time. In Nepal, Cole et al. [13] found that Tamang parents (i.e. a primarily Buddhist indigenous ethnicity) rebuked their children’s displays of anger, whereas Brahman parents (i.e. high-caste Hindus) responded to similar displays of anger with positive attention.

### Study context and objective

The aim of the current study was to evaluate the reliability and construct validity of a scale developed based on extensive ethnographic formative research in Nepal (i.e. the Disruptive Behavior International Scale-Nepal version (DBIS-N)), and to compare it with the Eyberg Child Behavior Inventory (ECBI) in assessing locally defined child behavior problems, and identifying children with poor functioning and parent-identified need for support. The primary purpose of the DBIS-N is to identify children with common behavior-related problems who might benefit from an indicated prevention or treatment intervention. The construct we sought to measure was behavior-related problems in children that were broadly related to disruptive, aggressive, and/or antisocial behaviors [14]. The DBIS-N is unique in that it was developed using local stakeholders’ input to prioritize items based on their perceived relevance and importance in the local context.

We hypothesized that: 1a) the items in the DBIS-N selected through exploratory factor analysis in a randomly selected development split sample would include at least one item identified from the local ethnographic research and exclude multiple domains included on international scales; 1b) the final version of the DBIS-N would be internally consistent (alpha> 0.70), have good inter-rater and test-retest reliability (intraclass correlation (ICC) > 0.60) [15], and demonstrate good fit indices in confirmatory factor analysis (see Methods sections for specific hypothesis). We also hypothesized that, compared with the ECBI, the DBIS-N would show incremental improvements in: 2a) identifying children reported to have locally identified behavior problems (via vignette nomination and a local behavior problem term); 2b) identifying children whose parents reported they had behavior problems and required support (for those problems); and 2c) identifying children with functional impairment, as measured by a local inventory of important functional roles. Finally, we explored rates of diagnoses in the sample population using a clinical interview and standard cut-offs for the ECBI.

## Methods

### Ethics approval

The study was approved by the Johns Hopkins University institutional review board and by the Nepal Health Research Council and was performed in accordance with the 1964 Declaration of Helsinki and its later amendments. Given the sensitivity of the research topic, written consent was provided by all adult study participants (i.e. children’s primary caregivers) and parents of child participants. Child participants (under age 18) provided verbal assent. A consent script was used to communicate the topic and purpose of the study, voluntary nature of participation, potential confidentiality risks to participants, and measures taken to protect confidentiality (including using a code on records instead of names and keeping all records locked). In order to ensure understanding, participants were asked to summarize the purpose and risks of participating in the study, and encouraged to ask questions.

### Study setting and population

The study was conducted in one of the Village Development Committees (VDCs; i.e. a small administrative area similar to a municipality) in Chitwan District in south-central Nepal. Chitwan District is a rural, primarily agricultural zone in the *Terai* (lowland) region near Nepal’s border with India.

### Participants

Participants for this study included the index children and their parents (or primary caregivers). The study included children (both boys and girls) between the ages of 5 and 15 years old residing in the study VDC. This age range was chosen due to considerations relating to school attendance, developmental stage, and family role definitions in the rural Nepali context: in Nepal, school attendance begins around age 5 and youth age 16–17 years have often completed secondary school (which finishes after grade 10), may be married, or may have left the community for further education or employment [16].

Subjects identified through sampling procedures (below) were included if they spoke Nepali, met age inclusion criteria (between 5 and 15 years old for index children; no age criteria for caregivers), and provided consent (adults) and assent (children).

### Sampling procedures

This study utilized a two-stage stratified sampling plan. Study recruitment and data collection took place between January and June 2015. The first stage utilized random sampling of households in order to achieve a probability sample of the population. A probability sample was desired in order to evaluate the discriminatory function of the tool in non-clinical settings in the local population, including low and medium levels of problem severity. In the first stage, households were randomly selected for screening (using computer generated random number) from a register of households in the study VDC that was previously obtained through a community enumeration survey of Chitwan District. A research assistant approached each identified household and spoke with an adult in the household to discuss participation in the study. If the adult agreed to participate, the research assistant explained the study procedures and discussed and obtained informed consent (adults) and assent (children), and proceeded to the second stage of sampling (see details below). If an adult was not present at the time of the visit, one additional attempt was made within one week of the initial attempt. If the adult declined participation, if there were no children living in the household, or if no adults were home after the second visit, the research assistant proceeded to the next household to the right (facing the house from the road) until a qualifying household was identified.

The second stage of sampling included stratification within households to achieve a weighted sample enriched for children with higher likelihood of DBDs. An enriched sample was desired in order to increase statistical power given the anticipated low prevalence rate of DBDs. In the second stage, a research assistant conducted screening of children age 5–15 residing within each selected household. The researcher read gender-specific vignettes of children with mild-moderate behavior problems (based on previous qualitative studies in Nepal [17–19]) to the head of the household and asked him or her to rate (on a 1–4 scale) the extent to which the description applied to each child, and whether they believed they needed support for that child. Children who met the description at least moderately well (i.e. rated 2, 3, or 4) were considered “screen positive”. One child was then selected from the household based on a “lottery” (i.e. drawing slips of paper from a bag) in which screen negative children were given one “chance” and screen positive children were given four “chances.”

We calculated the desired sample size with the goal of obtaining a sample sufficient to estimate the Receiver Operating Characteristic (ROC) curve (AUC) for the DBIS-N. While a priori sample size determinations for AUC are highly susceptible to assumptions about the performance of the test [20], Metz [21] has suggested that a sample size of 100 is generally sufficient to make a qualitative assessment of the utility of a test. Given the complexity and multiple assumptions involved, it is customary in validation studies to estimate sample size using comparison with previous validation studies with similar designs. In the case of assessment tools for DBDs, two of the most widely used assessment tools are the Strengths and Difficulties Questionnaire (SDQ) [22] and the Child Behavior Checklist (CBLC) [23]. Previous validation studies of the SDQ and CBLC have found that sample sizes of 199 and 201, respectively, were sufficient to establish optimal cutoff scores and convergent and discriminant validity with other scales and structured clinical assessments [22, 23]. Given that little is known about the epidemiology and use of assessment tools for DBDs in Nepal, we estimated that we would need to assess at least an additional 25% of the previous samples in case of low prevalence or unexpected measurement error. Thus, we aimed for a minimum sample size of 250 children.

### Sample characteristics

We screened 421 children from 268 households in the study community. Of these, 268 children (mean age 10.50 [standard deviation (SD) 2.84]; 42% female) were selected for the study and were evaluated with the DBIS-N and other instruments. We obtained DBIS-N ratings from a parent in 100% of subjects (99.8% of items complete). Additional sample characteristics are presented in Table 1.

**Table 1:** Study Sample Characteristics and Differences between Children Screened Negative vs. Positive for Behavior Problems

Characteristic	Screen negative^a^ (*n* = 137)	Screen positive^a^ (*n* = 131)	Overall Sample (*N* = 268)
	*n* (%)	*n* (%)	*n* (%)
Sex (% female)	53 (39.0)	59 (45.0)	112 (42.0)
Mean Age (SD)	10.5 (2.9)*	9.7 (2.7)*	10.2 (2.8)
Parents’ marital status			
Married	132 (96.4)	126 (96.2)	258 (96.3)
Divorced	0 (0)	1 (0.8)	1 (0.4)
Widowed	3 (2.2)	2 (1.53)	5 (1.9)
Separated	1 (0.7)	0 (0)	1 (0.4)
Re-married	1 (0.7)	2 (1.5)	3 (1.1)
Family type			
Nuclear family	81 (59.1)	74 (57.4)	155 (58.3)
Extended family	56 (40.9)	55 (42.6)	111 (41.7)
Caste/ethnicity			
Bahun/Chhetri	44 (32.4)	46 (35.1)	90 (33.6)
Dalit	8 (5.9)	5 (3.8)	14 (5.2)
Tharu	37 (27.2)	31 (23.7)	68 (25.4)
Kumal	24 (17.7)	34 (26.0)	58 (21.6)
Others	23 (16.9)	15 (11.5)	38 (14.2)
Religion			
Hindu	121 (88.3)	123 (93.4)	244 (91.0)
Buddhist	13 (9.5)	6 (4.6)	19 (7.1)
Christian	3 (2.2)	2 (1.5)	5 (1.9)
Parent working overseas	44 (32.1)*	61 (46.6)*	105 (39.2)

^a^Screening status based on initial screening using vignettes

*Significant (unadjusted) difference between screen-negative and screen-positive at *p* < 0.05 level (by t-test for continuous variables, chi-squared test for categorical variables)

### Study procedures: Data collection

For each selected child, a trained research assistant completed a demographic survey (17 brief questions) and the following assessments: the DBIS-N, the Child Functional Impairment Scale [24], the Ten Questions Plus [8], the Eyberg Child Behavior Inventory [25], and the emic nomination form (see below).

A psychosocial counselor then made a separate visit within 1–7 days to complete a semi-structured diagnostic clinical interview (see below). If available, mothers were the preferred respondents. The first 30 subjects (parents) were re-administered the DBIS-N by the same research assistant within 3–6 days of completing the initial data collection in order to evaluate test-retest reliability. Parents were the primary respondents for all instruments; children participated only in the semi-structured clinical interview. The total duration of both visits (combined) was approximately 90–120 min per family.

### Instruments

#### Disruptive behavior international scale—Nepal version (DBIS-N)

The DBIS-N was developed using a modified version of the scale development procedures outlined by DeVellis [26]. Complete study procedures for creating the initial pool of candidate items for the DBIS-N are described in another report [14] and are briefly reviewed here. This paper primarily reports on selection of items for the final scale and assessment of the scale’s reliability and construct validity. Candidate items were initially generated through: [1] local qualitative studies including free-listing, in-depth interviews, and focus group discussions with parents, teachers, community leaders and peer informants (*n* = 39 items) [14, 17, 19], and [2] a review of validated scales for behavior problems (*n* = 49 items), resulting in a total of 62 unique items. Candidate items were refined through cognitive testing with local stakeholders (through focus group discussions and individual interviews). Structured ratings were then used to assess the extent to which local stakeholders identified items as being important predictors of a “dark future” (Nepali: *andhiyaaro bhabishya*) and corresponding to “disobedient behavior” (Nepali: *badmaash*) [27]. Thirty items were dropped due to low ratings of importance and/or relevance.

The remaining 32 items were piloted in a group of 60 children. Based on these data, additional items were dropped based on poor comprehensibility (*n* = 2), low item-test correlation (*n* = 6), not acceptable to stakeholders (i.e. inappropriate to ask about) (*n* = 1), or extremely common or uncommon (*n* = 8) [26]; and 4 items were moved to an Adolescent Supplement based on low frequency in younger children (see [14] for full report on item reduction). The resulting problem scale included 16 items. Based on stakeholder feedback, 4 items assessing pro-social behaviors were added. All items were rated on a 0–3 scale based on frequency of occurrence (0 = “Never” to 3 = “Very Often”), with higher overall scores (range: 0–48) indicating more behavior problems. The current report evaluates the initially selected 20 items in a population-based sample.

#### Kiddie schedule for affective disorders and schizophrenia, present and lifetime (K-SADS-PL)

The K-SADS-PL is a semi-structured diagnostic clinical interview that yields categorical psychiatric diagnoses according to criteria outlined in the *Diagnostic and Statistical Manual (DSM)-III* and *–IV* [28]. The K-SADS-PL has been widely used in epidemiologic studies globally (c.f. [2]) and found to demonstrate good consensual validity with diagnosis by a psychiatrist in diverse settings, including Burundi [29] and Iran [30]. While not previously used in published studies in Nepal, the K-SADS-PL has been used for diagnosis of conduct disorder in India [31]. For this study, the Behavior Disorders Supplement (including subsections for ODD and CD) was administered. The questions were translated into Nepali, and minor adaptations were made to fit local conditions. One item (forced sex) was removed from the CD section based on feedback from local community members that it was inappropriate to ask about sexual behaviors in children. Each ODD and CD symptom was evaluated by the interviewer and rated on a 1–3 scale with 1 representing “not present,” 2 “subthreshold” level, and 3 “threshold” level. The interview also assesses duration and impairment related to the symptoms endorsed.

Clinical interviews were conducted by a psychosocial counselor with the child and (at least) one of the child’s primary caregivers. Psychosocial counselors are the main mental health providers in Nepal and have completed a 6-month standardized training course [5]. For this study, the two participating psychosocial counselors received additional training in interview techniques and use of the K-SADS-PL by the first author. Both counselors conducted practice interviews independently until their agreement reached 88% (kappa = 0.74).

#### Child functional impairment scale

Functional impairment was assessed using the Child Functional Impairment Scale (CFIS), a tool that has previously been used in Nepal to assess a child’s ability to complete 11 routine daily functions (e.g., household chores, homework, hygiene routines) expected of children in the study age range [24, 32]. Adult respondents report the extent to which a child’s ability to complete each expected daily function has been affected by problems related to his or her behavior. Each item is rated on a 0–3 scale (3 = difficulty “most of the time”). Total scores on the CFIS range from 0 to 33, with 33 representing the highest level of functional impairment.

#### Eyberg child behavior inventory

The Eyberg Child Behavior Inventory (ECBI), is a 36-item parent-report questionnaire that assesses child behavior problems using a 7-point scale to assess the frequency and a “yes/no” response to assess the current presence of specific problems [25]. The ECBI is scored according to “intensity” and “problem” domains, with “intensity” representing the summed numerical scores (range: 36–252, where higher numbers indicate greater “intensity” of behavior problems) and “problem” representing the total number of items that are reported as being a “problem” for the informant (range: 0–36, where higher numbers indicate a greater number of “problem” items) [25]. The ECBI has been widely used in a variety of cross-cultural settings, with reports indicating good reliability and validity in Asia [33, 34], Latin America [35, 36] and the Middle East [37]. To our knowledge, the ECBI has not been previously used in Nepal. The investigators translated and back-translated the items, and the author of the ECBI approved the final Nepali version.

#### Ten questions plus

The Ten Questions Plus is an 11-item parent-report screening tool for the presence of common neurodevelopmental disabilities, including delayed motor development, cognitive impairment, sensory deficits, and epilepsy [38]. Possible scores on the Ten Questions Plus range from 0 to 11, with higher scores indicating a greater number of neurodevelopmental problems. The Ten Questions Plus has previously been translated into Nepali and used in a neighboring region in the country [39].

#### Emic nomination form for Nepali behavioral syndromes

The emic nomination form for Nepali behavioral terms was developed for this study based on previous qualitative studies of behavior problems in the study area [19]. The form includes four common Nepali descriptors of children with behavior problems, including: *badmaash* (literal translation: naughty/disobedient); *chakchake* (restless/fidgety), *chucho* (mean/rude), and *bigrieko* (literal translation: “spoiled” or “broken”; refers to socially undesirable behavior). Parent respondents were asked to rate the extent to which the index child fits the description of each term using a 1–4 scale, with higher scores indicating a better “fit” with the label.

### DBIS-N item analysis and final scale development

We used a split-half sample to select items and validate findings. First, we divided the overall sample into two similarly sized groups using random number generation. In the first (i.e. “selection”) group, we conducted Exploratory Factor Analysis and eliminated items on the basis of: low loading (i.e. < 0.40) on factor 1 or 2, complex factor loading structures (i.e. > 0.32 on more than one of the first 3 factors), or low item-rest correlation (< 0.30) [40]. Items were eliminated sequentially (based on worse performance) and the overall scale reliability was checked using Cronbach’s alpha after each step to ensure the reliability was not negatively affected.

After poorly fitting items were dropped, we conducted Confirmatory Factor Analysis in the second (“validation”) group and checked item factor loadings and model fit indices. Good fit was indicated by Root Mean Square Error of Approximation (RMSEA) < 0.06, Comparative Fit Index (CFI) > 0.95, and Non-normed Fit Index (NNFI) > 0.95 [41]. Dimensionality of the scale was evaluated using visual inspection of the scree plot, eigenvalues, and parallel analysis using the *paran* package in Stata.

#### Reliability

After we selected items for the final version of the DBIS-N, we evaluated multiple aspects of reliability in the final scale. Cronbach’s alpha was used to assess internal consistency of items on the DBIS-N. Inter-rater reliability was assessed by evaluating the consistency of ratings taken by two research assistants interviewing the same parent. For test-retest reliability and inter-rater reliability, intra-class correlation (ICC) and Pearson’s correlation coefficient were calculated.

### Comparison of emic and etic assessment methods

#### Criterion validity and classification accuracy

Given the primary goal of this project to evaluate the measurement of locally meaningful constructs related to child behavior problems, and in the absence of “gold standard” assessment for these constructs, we used two criteria: local nominations of constructs using a variety of tools and a vignette-based assessment. “Cases” were those who were identified (aka “nominated”) as *badmaash* using an emic-based tool and also had functional impairment in locally identified domains of child functioning, as indicated by an elevated score (>75th percentile) on the CFIS. The second criterion was children identified as having behavior problems in the vignette-based assessment whose parent also stated that they were in need of support. We then evaluated criterion validity by comparing classification accuracy on these two criteria of the DBIS-N, the ECBI (an externally-derived scale) and the KSADS-PL, a structured clinical interview (KSADS-PL).

We used Area Under the Curve (AUC) (using *roctab* in Stata) to compare classification accuracy between assessment methods (i.e. DBIS-N, ECBI, KSADS-PL) for each emic domain. Given our unanticipated finding of very low rates meeting diagnostic criteria for ODD and CD on the KSADS-PL, and elimination of one of the items for CD, we used alternate (i.e. slightly lower threshold) criteria for diagnosis for analytic purposes (details below).

#### Incremental validity

We also assessed incremental validity using progressive multiple logistic regressions on both local criteria [42]. As independent variables, we included demographic characteristics associated with behavior problems identified through univariate logistic regression (i.e. age and sex) and developmental delays (according to the Ten Questions Plus). We considered the DBIS-N to show incremental validity if, when it was added to the model including ECBI as a variable, its beta was statistically significant at the alpha = 0.05 level, indicating an independent contribution to explaining variability in the local criteria above and beyond the ECBI. We also examined change in R^2^ before and after the DBIS-N variable was added.

#### Statistical analysis

Statistical tests for the validity study were performed using Stata 12.0 [43]. We used Pearson’s correlation coefficient to evaluate linear relationships between interval variables. We used Spearman correlations to evaluate correlations between variables in which at least one variable was ordinal. We used pairwise deletion for observations with missing data when calculating intra-class correlations (ICC) and Pearson’s correlation.

## Results

### DBIS-N item analysis and final scale development

Based on analyses from the development sample, we dropped six items due to low item-rest correlation (*n* = 4), low loading on factor 1 (*n* = 4), and cross-loading on factors 1 and 2 (*n* = 3). After dropping the six items, Cronbach’s alpha in the development sample increased slightly from 0.81 to 0.82. The revised scale included ten behavior problem items, including three locally derived items, one item taken directly from international scales, three items locally adapted from international scales, and three items from both local interviews and international scales (see Table 2. In the validation sample, all items loaded > 0.40 on factor 1, there were no cross-loadings > 0.30 on factors 2 or 3, and Cronbach’s alpha was 0.84. The remaining results (below) are from the entire sample.

**Table 2:** Factor loadings for items in the final version of DBIS-N (total sample)

Item	Source	F1	F2	F3	Uniqueness
5 Boldly disobedient	I+	0.69	−0.26	0.07	0.45
6 Angry over small things	I+	0.63	−0.10	− 0.17	0.56
8 Curses	L	0.52	0.22	0.09	0.68
9 Lies	B	0.42	0.21	−0.01	0.78
10 Fails to follow instructions from elders	I+	0.65	−0.21	0.07	0.53
11 Fights with other children	B	0.53	0.07	0.19	0.68
13 Spends time with children who do bad things (“walks in bad circle”)	L	0.55	−0.02	0.04	0.69
14 Deliberately annoys others	I	0.60	−0.08	−0.11	0.62
15 Argues with elders	L	0.60	0.13	−0.06	0.62
18 Talks back to adults	B	0.65	0.20	−0.07	0.53

Abbreviations: *L* Local interviews, *I* International scales, *I+* local adaptation of common international item, *B* Both (i.e. found in both international scales and local interviews)

### DBIS-N reliability and factor structure

The DBIS-N had good internal consistency (Cronbach’s alpha: 0.84). The test-retest ICC was 0.93 and *r* = 0.93 (i.e. very strong). ICC of the inter-rater reliability (different RAs interviewing same parent) was 0.62 and *r* = 0.68 (i.e. strong).

Exploratory factor analysis revealed a unidimensional factor structure for the DBIS-N (eigenvalues: factor 1 = 3.48, factor 2 = 0.28). Additional analysis of the number of factors using parallel analysis (*paran* package in Stata) with principal components analysis yielded similar results (adjusted eigenvalue for factor 1: 3.83 and factor 2: 0.68; see Additional file 1: Figure S1). (Item factor loadings are listed in Table 2.)

### DBIS-N score distributions

The mean total DBIS-N problem scores was 4.75 (SD 4.15). DBIS-N scores were skewed, with 56% of children scoring 4 or less. There was no difference between mean scores of girls and boys (*t*(264) = 0.03, *p* = 0.98). Total problem scores decreased with increasing age (β = − 0.27, *p* = 0.002).

### Comparison of emic and etic assessment methods

#### Emic assessments

According to the locally derived behavior problem vignette, 49% of children were rated by parents as having behavior problems; among those who screened positive, 82% of parents indicated that they “needed support” for their child’s behavior problems. Using the emic nomination form, 26% of children were identified by parents as “definitely” *badmaash*.

#### ECBI

The ECBI had good internal consistency (Cronbach’s alpha: 0.91). In exploratory factor analysis, three items had low loadings across all factors, six items had complex loadings, and one item loaded only on factor 2. These items dealt with timeliness, carelessness with toys, stealing, problems with attention and concentration, “difficulty entertaining self alone”, and enuresis. (Additional file 2: Table S1 presents summary scores from all primary assessment scales.)

#### Clinical interviews (K-SADS-PL)

Only 1 child (0.4%) met DSM-IV diagnostic criteria on the K-SADS-PL for ODD, and 2 (0.8%) met criteria for CD. Given the very low prevalence of children meeting full criteria for ODD or CD, we also evaluated subthreshold symptoms (i.e. presence of symptom below “threshold” level for diagnostic criteria as defined in K-SADS-PL) of both disorders on the K-SADS-PL. Two hundred five (77%) children had at least one symptom of ODD at the “subthreshold” level. The mean number of ODD symptoms endorsed at the subthreshold level was 2.86 (SD 2.59), and subthreshold symptoms were a good predictor of ODD-related impairment as ascertained using the K-SADS-PL (OR for impairment with each additional subthreshold symptom = 1.63 (95% confidence interval (CI): 1.37–1.93, *p* < 0.001). Eighty-four (31%) children had at least one symptom of CD at the “subthreshold” level. The mean number of CD symptoms endorsed at the subthreshold level was 0.74 (SD 1.45), and subthreshold symptoms were a good predictor of CD-related impairment as ascertained by the K-SADS-PL (OR for impairment with each additional subthreshold symptom = 2.28 [95% CI: 1.55–3.35, *p* < 0.001]).

### Comparison of assessment methods

Comparisons of etic and emic assessments, including the DBIS-N, are presented in Table 3. Compared with the ECBI, the DBIS-N was more strongly correlated with nomination on the locally derived vignette (rho = 0.57 vs. 0.49 for the DBIS-N and ECBI, respectively) (z = 1.28, 2-sided *p* = 0.20), while the scales correlated similarly with nominations of local behavior problem term *badmaash* (rho = 0.54 vs. 0.53; z = 0.16, 2-sided *p* = 0.87). The DBIS-N was less strongly correlated with functional impairment (as measured by the CFIS) compared with the ECBI (*r* = 0.58 vs. 0.68; z = − 1.91, *p* = 0.06).

**Table 3:** Correlations between Parent Report Measures: Convergent & Discriminant Validity

Measure	1^a^	2^b^	3^b^	4	5	6	7	8
Locally-derived behavior problem measures (convergent validity)								
1 DBIS-N (parent report)	*–*							
2 Vignette-based nomination^b^	*0.59*	–						
3 Behavior problem term nomination^b^ (*badmaash* [naughty/disobedient])	*0.57*	0.55	–					
Externally-derived behavior problem measures (convergent validity)								
4 ECBI	*0.84*	0.53	0.53	–				
5 ODD symptoms on K-SADS-PL^c^	*0.58*	0.39	0.41	0.59	–			
6 CD symptoms on K-SADS-PL^c^	*0.44*	0.31	0.36	0.45	0.60	–		
Functional impairment (convergent validity)								
7 Functional impairment (CFIS)	*0.63*	0.36	0.30	0.68	0.35	0.32	–	
Different constructs (discriminant validity)								
8 Ten Questions Plus (total score)	*−0.27*	− 0.01	− 0.09	−0.34	− 0.26	−0.19	− 0.38	–

^a^Column “1” indicates the study instrument (DBIS-N)

^b^Correlation calculated using Spearman’s rank-sum correlation coefficient for ordinal variables

^c^Calculated using number of ‘subthreshold’- and ‘threshold’-level symptoms endorsed

Abbreviations: *DBIS-N* Disruptive Behavior International Scale—Nepal version, *ECBI* Eyberg Child Behavior Inventory, *K-SADS-PL* Kiddie-SADS-Present and Lifetime version, *CFIS* Child Functional Impairment scale, *ODD* Oppositional Defiant Disorder, *CD* Conduct Disorder

### Criterion validity

Classification accuracy and AUC were similar and good for the ECBI and DBIS-N, but substantially poorer for KSADS-PL, on both emic criteria: 1) nomination for *badmaash* (with functional impairment) and 2) vignette-based nomination (with parent-reported need for support) (see Table 4).

### Incremental validity

Based on univariate regression analyses, we included sex, gender, and developmental delays in our multivariate logistic regression on both emic criteria. For *baadmash,* DBIS-N was statistically significant (*p* = .01), ECBI no longer remained significant (*p* = 0.18) and the model R^2^ increased from 0.27 to 0.31. For vignette-based nomination, DBIS-N was statistically significant (*p* < 0.001) and the model R^2^ increased from 0.27 to 0.32 (see Table 5).

**Table 4:** Area Under the Curve and Classification Accuracy for the DBIS-N, ECBI, and KSADS-PL using two emic assessments as criteria

Local Construct (Criterion)	DBIS-N		ECBI		K-SADS-PL	
	AUC (95% CI)	Classification Accuracy	AUC (95% CI)	Classification Accuracy	AUC (95% CI)	Classification Accuracy
Vignette-based behavior problem*	0.83 (0.78–0.88)	76.0%	0.83 (0.78–0.88)	75.2%	0.49 (0.42–0.56)	54.3%
*Badmaash* (naughty/ disobedient)**	0.85 (0.77–0.93)	90.0%	0.85 (0.78–0.91)	88.8%	0.49 (0.38–0.60)	88.1%**

Abbreviations: *DBIS-N* Disruptive Behavior International Scale-Nepal, *ECBI* Eyberg Child Behavior Inventory, *K-SADS-PL* Kiddie SADS Present and Lifetime, *AUC* Area Under the Curve

*Children nominated by their parents as having locally defined behavior problems based on vignette description and affirmation of need for support. ** Children identified by their parents as being “definitely” *badmaash* (translation: naughty/disobedient) and meeting locally defined criteria for functional impairment (i.e. CFIS > 9)

**Highest classification accuracy was at the maximum score, which yielded 0% sensitivity, and 100% specificity

**Table 5:** Incremental validity assessment using multiple logistic regression analysis

Dependent Variable	Model 1				Model 2		
	Independent variable	B (SE)	*p*	Total variance explained (model) (*R*^*2*^)	B (SE)	*p*	Total variance explained (model) (*R*^*2*^)
Vignette-based behavior problem	Age	0.02 (0.06)	0.72	0.27	0.31 (0.06)	0.60	0.32
	Female sex	0.50 (0.32)	0.11		0.37 (0.26)	0.26	
	Dev. delays	−0.30 (0.16)	0.05		−0.33 (0.05)	0.05	
	ECBI	0.09 (0.01)	< 0.001		0.05 (0.02)	0.002	
	DBIS-N	–			0.32 (0.08)	< 0.001	
*Badmaash* nomination	Age	−0.06 (0.08)	0.46	0.27	−0.07 (0.38)	0.38	0.31
	Female sex	−0.80 (0.50)	0.11		−0.97 (0.06)	0.06	
	Dev. delays	0.05 (0.15)	0.72		0.06 (0.16)	0.70	
	ECBI	0.06 (0.01)	< 0.001		0.02 (0.02)	0.18	
	DBIS-N	–			0.21 (0.09)	0.01	

Abbreviations: *ECBI* Eyberg Child Behavior Inventory, *DBIS-N* Disruptive Behavior International Scale-Nepal

## Discussion

This study assessed the reliability and construct validity of the DBIS-N—a scale developed using ethnographic research in Nepal—and compared it with a widely used Western-derived scale (ECBI) in assessing locally defined child behavior problems. Findings from our study demonstrate the reliability and construct validity of the DBIS-N. Using parent-reported nominations for locally defined child behavior problems as criteria, the ECBI and DBIS-N showed similar AUC and classification accuracy, while the DBIS-N added predictive value above the ECBI, supporting its incremental validity. While the ECBI was a better predictor of functional impairment, ten of 36 items were problematic in factor analysis. Due to the very small number of cases of ODD and CD identified through clinical interviews, we were unable to assess the criterion validity of the DBIS-N using clinical diagnosis as planned. Below, we discuss key findings, implications for practice, study limitations, and considerations for utility of the DBIS-N vs. externally-derived scales in low-resource settings like Nepal.

While both scales showed good internal consistency and were correlated with functional impairment, the DBIS-N performed better than the ECBI in identifying local idioms of child behavior problems. This difference may reflect the relevance of individual items to local concerns and consistency with culture-specific values for child behavior in Nepal. The items in the final version of the DBIS-N were selected through a process of ethnographic inquiry, reviewing existing scales, item evaluation by stakeholders, and factor analysis and consisted of themes related to anger, defiance, and relational problems, especially regarding elders. In contrast, items in the ECBI were developed in Western contexts and translated into Nepali. Factor analysis of the ECBI revealed problematic loading patterns in ten of the 36 items. Problematic items from the ECBI largely focused on timeliness, carelessness with belongings, problems with attention and concentration, and “difficulty entertaining self alone”—domains that did not pertain to areas of concern in prior studies of local stakeholders [17, 19]. Of particular importance for cross-cultural assessment, there were no items in the ECBI that specifically addressed the importance of respect for elders, which is one of the most important behavioral norms for children in most of the world’s cultures [44, 45]. The most closely related items in the ECBI focused on following directions from parents and there were no items that addressed relationships with adults other than parents. Taken together, these differences in content are reflective of prevalent multigenerational household composition in Nepal and widely shared values of respect for elders, while also reflecting a more socio-centric value system with less concern for individual time (e.g., “entertaining self alone”) or timeliness [46, 47].

Our study contributes to the field of cross-cultural scale development in child mental health by offering a systematic procedure to incorporate local concerns and problem manifestations into measurement scales. Developing valid and reliable tools for assessment for use across cultures and settings was identified as a top priority for global mental health in a major cross-national priority-setting effort [4]. Current widely used scale development procedures (c.f. 26) rely primarily on academic experts to generate and select candidate items for scales. In cross-cultural scale development, local stakeholders are typically involved in later stages of checking the coherence of item translation and phrasing (i.e. “cognitive interviewing”) [5, 48]. Our study provides an example of earlier, more extensive, systematic engagement with local stakeholders to first understand the context of the mental health problem (using ethnographic inquiry), and then to generate items locally (drawn from interviews and free-listing), and evaluate their relevance to the local context (through ratings and interviews)—all prior to the cognitive interviewing stage. Given our findings that this process resulted in a valid and reliable scale with incremental validity over a widely used translated scale, our procedures may be used in future cross-cultural scale development efforts as a systematic approach to address concerns about local salience of symptoms and disorders and to reduce the risk of category fallacy. Ensuring the local relevance of disorders and indications for interventions represents an important step for avoiding harm and promoting engagement with vulnerable children and families in low-resource settings [5, 7].

An important finding in our study was the small number of cases identified using the K-SADS-PL clinical interview, despite targeting an enriched population. The low rate of qualifying symptoms identified may reflect a low rate of child behavior problems in the study population, less relevant diagnostic criteria in this population, social desirability bias by the respondent (which may vary by ascertainment method), or a different calibration for distinguishing between sub-threshold and “threshold” symptoms by the clinical interviewers. Compared to samples of children of similar ages in the U.S. [49] and Norway [50], the Nepali children in this study also scored somewhat lower on the problem intensity scale of the ECBI, but not enough to explain the extremely low prevalence of diagnoses. These cross-national comparisons support the possibility of different rates of problem behavior, social desirability bias, different parental thresholds [12], or a combination of contributing factors.

Alternatively, the low rate of diagnoses may reflect limitations of the K-SADS-PL with culture-specific behaviors that fail to capture children with behavior problems in contexts that differ from those in which the instrument was developed. This represents a challenge for validation when the clinical interview is also biased toward culture-specific behaviors. To address the resulting limitation for assessing criterion validity, we used any symptom endorsement on the K-SADS-PL (i.e. including at the “subthreshold” level), which resulted in weak to moderate correlations with the DBIS-N, functional impairment, and other assessments of behavior problems. The finding of poor convergence with clinical symptom assessments of ODD and CD is similar to a previous scale development effort for behavior problems in another low-income country setting (Ng et al., 2014). Together, these findings suggest that problems in using structured clinical interviews (such as K-SADS-PL) for behavior problems may be related to the “problem” threshold applied and to the range of behaviors surveyed. These differences highlight the importance of evaluating alternative construct definitions of behavior problems (other than those used in structured clinical interviews developed in Western contexts) and/or considering alternative methods of case ascertainment in low-income country contexts.

A strength of our study is that it is one of few validation studies of a scale for child behavior problems performed in a low-income, non-Western country setting that utilized a population-based probability sample. Compared with commonly used practices (e.g. comparing an “extreme” clinical group likely to have the condition of interest based on attendance in a clinic or nomination by community members), a probability sample allowed us to assess how the instrument functions in actual screening settings in which pre-test probability is unknown. Our two-stage sampling involving initial screening and probability sampling had the benefits of both an enriched sample (therefore increasing statistical power) and a sample that represents much of the demographic and clinical diversity of the population. Therefore, our estimates of classification accuracy are more likely to approximate the functioning of the instrument in actual practice situations evaluating children with a wide range of problem severity.

### Implications

Several factors are important for selecting useful tools for mental health screening. In addition to their psychometric soundness (e.g. reliability and factor structure), tools must measure a construct that is meaningful to stakeholders involved, be accurate in identifying children who could benefit from a service, and meet practical criteria for implementation [5]. Our findings above have demonstrated the reliability of the DBIS-N as well as its ability to identify children with locally meaningful idioms for child behavior problems whose parents indicated their need for support.

In addition, the DBIS-N addresses practical needs for screening tools in low-resource settings. Compared with the ECBI and other widely used international tools, the DBIS-N is brief (i.e. 10 questions vs. 36 (ECBI) or more than 100 (Child Behavior Checklist (CBCL) [51]), therefore helping to address time constraints in busy healthcare or educational settings. While the DBIS-N is freely available, most widely used behavior problem inventories—including the ECBI and the CBCL—are proprietary and involve per use expenses, making them impractical for widespread use in low-resource settings. Thus, the DBIS-N offers a brief, free scale in the Nepali language with local evidence of validity and is well suited for use in identifying symptomatic children for prevention (“targeted”) or treatment interventions in Nepal. Specifically, the DBIS-N could be used to screen children in school or community settings for inclusion in psychosocial interventions for behavior problems [52].

Finally, the item generation, selection, and validation processes used to create the DBIS-N may be broadly applicable in creating locally valid tools for measuring child behavior problems in other cross-cultural settings. Systematically incorporating local stakeholder input for generating and selecting items, as outlined in our procedures, is likely to enhance understandability and reduce the likelihood of category fallacy when creating locally adapted measurement tools for child behavior problems.

### Limitations

The small size of the initial development sample [14] may have increased the possibility of overlooking behavioral concerns of segments of the population, and may have led us to eliminate less frequently endorsed, but important, symptoms from the final tool. On the other hand, our study is one of only a few tool validation studies in global mental health to use a multi-stage design with a development sample. The clinical interviews in the validation study may also have been limited by reliance on non-specialist interviewers, which may have increased variability due to error and decreased comparability with international studies using specialist interviewers. The choice to use trained non-specialist interviewers was based on actual practice conditions in Nepal, where psychologists with advanced degrees are extremely rare [53]. The use of trained non-specialist interviewers is also consistent with other validation studies in Nepal [5] and other LMIC [11]. We also attempted to compensate for the lack of specialist clinical interviewers by triangulating findings with multiple evaluation methods, including nomination based on emic categories, standard international scales, and assessment of functional impairment, as proposed by Bolton [54]. Finally, our study is limited by reliance on reported symptoms. Our findings would have been strengthened by comparison with prolonged direct observations of children’s behavior in home and school settings; however, direct observations posed some ethical and significant practical barriers.

## Conclusions

This study supports the reliability and construct validity of the DBIS-N, a behavior problem measurement tool developed using ethnographic research and local stakeholder ratings to account for behavioral norms in non-Western cultural settings. To our knowledge, this is the first measure of child behavior problems developed based on empirical observations and validated in a population-based sample in South Asia, and one of the first meeting these criteria in a non-Western setting.

While both the DBIS-N and the ECBI had high classification accuracy for local idioms for behavior problems, the DBIS-N had a more coherent factor structure and added predictive value above the ECBI. Items from the DBIS-N were more consistent with cultural themes identified in qualitative research, whereas multiple items in the ECBI that did not fit with these themes performed poorly in factor analysis. In conjunction with practical considerations such as price and scale length, our results lend support for the utility of the DBIS-N for the assessment of locally prioritized behavior problems in Nepal. The use of systematic procedures with local stakeholder participation may represent a widely applicable process for developing locally adapted scales in other non-Western cultural settings.

## Additional files

Additional file 1: Figure from parallel analysis (using paran package in Stata) to identify number of factors to retain. (JPG 96 kb)

Additional file 2: Mean scores and frequencies for primary assessment scales. (PDF 73 kb)

## Abbreviations

AUC: Area under the curve
CBCL: Child behavior checklist
CD: Conduct disorder
CFI: Comparative fit index
CFIS: Child functional impairment scale
CI: Confidence interval
DBD: Disruptive behavior disorders
DBIS-N: Disruptive behavior international scale—Nepal version
DSM-5: Diagnostic and statistical manual of mental disorders, Fifth Edition
ECBI: Eyberg child behavior inventory
ICC: Intraclass correlation
K-SADS-PL: Kiddie schedule for affective disorders and schizophrenia, present and lifetime
LMIC: low- and middle-income countries
NNFI: Non-normed fit index
ODD: Oppositional defiant disorder
RMSEA: Root mean square error of approximation
SD: Standard deviation
VDC: Village development committee
