# Supplementary figures and images for: Validation of a cross-cultural instrument for child behavior problems: the Disruptive Behavior International Scale – Nepal version
Source: BMC Psychol. 2018 Nov 3;6:51. doi: 10.1186/s40359-018-0262-z (PMC6215604; doi:10.1186/s40359-018-0262-z)

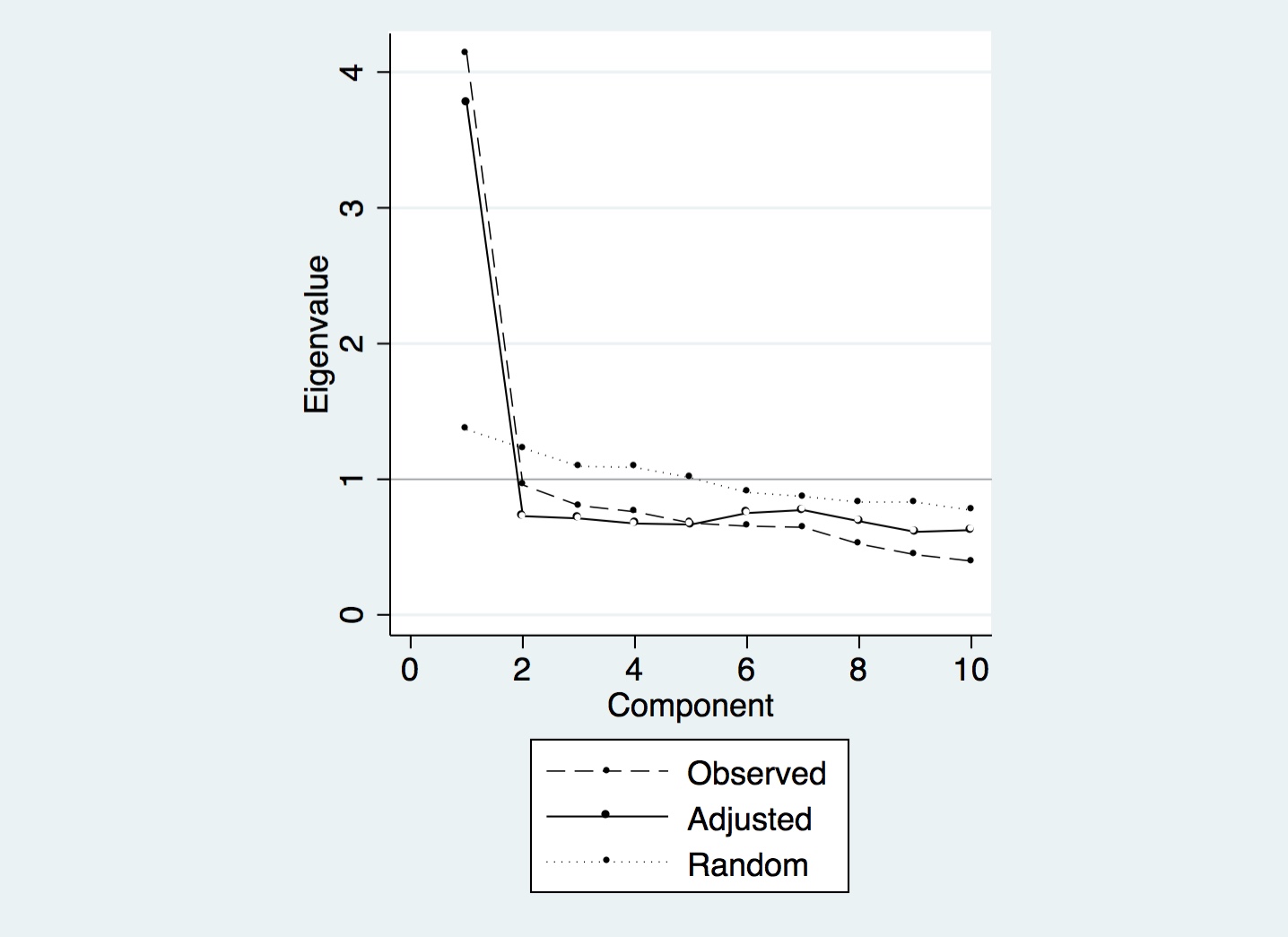

Supplement: Supplementary file 1 — Figure from parallel analysis (using paran package in Stata) to identify number of factors to retain. (JPG 96 kb) [file 40359_2018_262_MOESM1_ESM.jpg]
