# Supplementary material for: Validation of a cross-cultural instrument for child behavior problems: the Disruptive Behavior International Scale – Nepal version
Source: BMC Psychol. 2018 Nov 3;6:51. doi: 10.1186/s40359-018-0262-z (PMC6215604; doi:10.1186/s40359-018-0262-z)
Supplement: Supplementary file 2 — Mean scores and frequencies for primary assessment scales. (PDF 73 kb) [file 40359_2018_262_MOESM2_ESM.pdf]

Supplementary Table 1

Mean scores and frequencies for primary assessment scales

Measure	*N*	Mean	SD	Range	Skew
DBIS-N	267	4.75	24	0-24	1.48
ECBI	268	59.24	168	36-168	1.93
K-SADS-PL ODD*	268	2.86	9	0	0.64
K-SADS-PL CD*	268	0.74	7	0	2.32
Functional impairment (CFIS)	268	4.79	22	0-22	1.18
Ten Questions Plus	268	1.22	1.2	0-7	2.41

* Number of ‘subthreshold’- and ‘threshold’-level symptoms endorsed

Abbreviations: SD: Standard deviation; DBIS-N: Disruptive Behavior International Scale—Nepal version; ECBI: Eyberg Child Behavior Inventory; K-SADS-PL: Kiddie-SADS-Present and Lifetime version; CFIS: Child Functional Impairment scale; ODD: Oppositional Defiant Disorder; CD: Conduct Disorder
